# Newborn screening in Germany

**DOI:** 10.1515/medgen-2022-2119

**Published:** 2022-05-07

**Authors:** Holger Tönnies, Uta Nennstiel

**Affiliations:** Geschäftsstelle Gendiagnostik-Kommission, Robert Koch-Institut, Nordufer 20, 13353, Berlin, Germany; MPH, Bayerisches Landesamt für Gesundheit und Lebensmittelsicherheit, Veterinärstr. 2, 85764, Oberschleißheim, Germany

Newborn screening (NBS) from dried blood spot cards was first implemented in the late 1960s and is considered the most successful secondary prevention in childhood. In most countries, screening is offered to all newborns as a public health measure. Any screening program, in addition to being of great benefit to the person affected by a target disease, can also cause harm through false positive (abnormal) findings. These constitute a high burden on the health care system and short- or long-term psychosocial stress on families (worrying, vulnerable child syndrome). Detection of mild or unclear severity of the disease can lead to overtreatment and stigmatization and should be avoided whenever possible. For these reasons, new target diseases for NBS must be selected very carefully and in consideration of the screening criteria of Wilson and Jungner [1] updated by Andermann [2].

In Germany, expanded newborn screening (ENS) for inborn errors of metabolism and endocrinopathies was introduced to the benefits catalog of the statutory health insurance funds in July 2005 and has since been added to. ENS in Germany currently includes 19 target disorders consisting of 13 metabolic disorders, 2 endocrinopathies, cystic fibrosis (CF), severe combined immunodeficiencies (SCID), sickle cell disease (SCD) and spinal muscular atrophy (SMA). The basic features of the ENS procedure are regulated in §§ 13 to 42 of the Children’s Guideline on the Early Detection of Diseases (“Children’s Guideline”) adopted by the Federal Joint Committee (G-BA) pursuant to § 26 of the German Social Code, Book V (Gemeinsamer Bundesausschuss, SGB V). In addition, ENS is subject to the regulations of the Genetic Diagnosis Act (GenDG), which came into force on 01.02.2010.

This issue of medizinischegenetik focuses on five topics in the context of newborn screening in Germany:

– Legal aspects of NBS in Germany,
– Challenges and suggestions for genomic NBS (gNBS) including ELSA,
– Potential and Limitations of second-tier strategies in NBS,
– Registries for rare conditions as an important public-health measure,
– Concept of a multidisciplinary, population-based screening program using routine genetic testing.

In their introductory article “**Legal aspects of newborn screening**” Rosenau and Steffen focus on legal aspects of newborn screening in Germany. Genetic diagnostics and newborn screening are regulated by the Gendiagnostikgesetz (GenDG). The authors delineate, that since the act came into force, some legal questions have been resolved over the years, but, however, new questions arose over time, which now need to be addressed. As one example, the handling of heterozygous information detected by genetic analysis in newborn screening is discussed.

Newborn screening programs are considered among the most effective public health programs of the 20th and 21st centuries. An overview of the most pressing questions to be considered when implementing genomic newborn screening (gNBS) is provided in Dikow et al. “**From newborn screening to genomic medicine: challenges and suggestions on how to incorporate genomic newborn screening in public health programs**”. They state, that the concept of gNBS is methodologically feasible and provides highly relevant information for newborns and their families. They target the question, if parents should get the right to receive relevant genetic data from gNBS, if they request it. The authors conclude, that current NBS will not be replaced by gNBS in the near future. Implementing gNBS bears substantial implications for societal perception of health risk and values, which go beyond the individual’s or family’s health care. Therefore, a broad discussion in the general public is needed.

Every additional target disorder in newborn screening inevitably leads to an increase of false-positive NBS results. And: NBS using conventional marker metabolites alone is associated with a relatively low specificity, leading to a high number of false-positive NBS results. To circumvent unnecessary concerns for parents and newborns and to increase specificity of NBS for disorders without a highly specific primary NBS marker, second-tier strategies have been developed. Gramer and coauthors are focusing on this topic in their manuscript “**Second-tier strategies in newborn screening – potential and limitations**”. They show, that in addition to biochemical second-tier markers, also genetic second-tier or third-tier analyses are a promising approach to improve NBS results and maintain the high acceptance of NBS by families.

Registries for rare conditions should be considered as an important public-health measure and they should be adequately institutionalized. Hammersen and colleagues contribute valuable data on two long term existing registries in Germany in their report “**Twenty years of newborn-screening for congenital adrenal hyperplasia and congenital primary hypothyroidism – experiences from the DGKED/AQUAPE study group for quality improvement in Germany**”. Both the CH and the CAH registry include data from a large number of patients treated at participating expert centers, containing real-life data on anthropometric data, laboratory results, treatment, and long-term outcome. These registries allow research studies based on real-life data, often with direct clinical implications. They show that reliable information on long-term follow-up of patients detected in newborn-screening programs is essential to assess the benefit of newborn screening programs and identify gaps and suboptimal care. In Germany, there is no registry addressing all screening diagnoses.

With a prevalence of 1:250, Familial Hypercholesterolemia (FH) is the most frequent monogenic disorder (prevalence 1:250) in the general population, but actually not part of the ENS in Germany. However, early diagnosis during childhood would enable pre-emptive treatment, thus reducing the risk of severe atherosclerotic manifestations later in life. Sanin and colleagues present in their manuscript “**Population-based screening in children for early diagnosis and treatment of familial hypercholesterolemia: design of the VRONI study**” a study-based multidisciplinary screening program for FH in children aged 5 to 14 years, involving diverse branches within the health care sector as well as logistic and legal support by a scientific infrastructure. In this proof-of-concept study, routine genetic testing for the early detection of FH in children is used. They state, that by routine screening of children and reverse cascade screening of first-degree relatives, FH can be diagnosed before the onset of cardiovascular events, enabling preventive treatment. VRONI also provides important data regarding the trend to a personalized approach, such as using genotype, lifestyle and environmental factors for tailoring treatment.

We hope that the five scientific articles in this special issue will give you good insights into the field of newborn screening in Germany wish you a stimulating read.

We sincerely thank all the dedicated authors, coauthors, reviewers, and organizers of this issue who contributed to its success.
